# Proton therapy achieves high-dose tumor control with organ preservation in complex metastatic adenoid cystic carcinoma: a case report of a refractory patient with 16 pulmonary metastases

**DOI:** 10.3389/fonc.2025.1669420

**Published:** 2025-11-06

**Authors:** Zejun Lu, Xuanrong Zhang, Zhiqiang Jiang, Haiqiang Chu, Lei Li, Zishen Wang, Yingrong Xie, Bin Ren, Jingbo Kang

**Affiliations:** Department of Radiation Oncology, Hebei Yizhou Cancer Hospital, Baoding, Hebei, China

**Keywords:** adenoid cystic carcinoma, proton, refractory, metastases, dosimetric

## Abstract

Adenoid Cystic Carcinoma (ACC) is characterized by its aggressive nature, high propensity for perineural invasion, and significant risk of distant metastasis, particularly to the lungs. Therapeutic options for locally advanced or metastatic ACC are limited, and conventional radiotherapy is often constrained by dose limitations for multifocal disease, leading to inadequate treatment. A 54-year-old male ACC patient, who had recurred after three prior surgeries, presented with PET/CT-confirmed bilateral cervical lymph node and multiple bilateral pulmonary metastases. Given the multifocal metastases and the critical need for sparing organs at risk (lungs, heart, esophagus), which rendered photon radiotherapy unable to meet the required dose constraints, proton therapy was employed with the following dose prescriptions: for pulmonary metastases: CTV 50 Gy(RBE) in 15 fractions, GTV 60 Gy(RBE) in 15 fractions; for the cervical lesion: GTVnd 70 Gy(RBE) in 28 fractions, CTVnd 66 Gy(RBE) in 28 fractions, CTV 50.4 Gy(RBE) in 28 fractions. Follow-up PET/CT post-treatment demonstrated complete resolution of some bilateral pulmonary metastases, with marked reduction in size and decreased metabolism in the remaining nodules. The metastatic cervical lymph nodes also showed reduced volume and metabolic activity. No adverse events exceeding Grade 2 occurred during the treatment course. This case demonstrates that proton therapy is highly suitable for multifocal ACC metastases, especially multiple small pulmonary nodules. Through its precise dose delivery, it enables high-dose irradiation (GTV 60–70 Gy(RBE)) to targets while significantly sparing normal organs. This approach represents a viable strategy for complex cases where conventional radiotherapy is contraindicated. It aims to delay disease progression and achieve organ preservation in refractory ACC.

## Introduction

ACC is a rare malignant tumor of the salivary glands, accounting for 1%–2% of head and neck malignancies (1). Its pathological hallmarks include perineural invasion, a high local recurrence rate, and a propensity for delayed pulmonary metastasis. Indeed, distant metastases eventually develop in approximately 40%–60% of advanced patients, most commonly in the lungs (2). Although surgery combined with adjuvant radiotherapy can improve local control, systemic options for metastatic ACC are severely limited. Chemotherapy yields response rates of less than 30% (3). Furthermore, conventional photon radiotherapy is significantly challenged by multifocal metastases, as dose constraints for organs at risk often compel suboptimal tumor doses, resulting in local failure (4). In recent years, proton beam therapy (PBT) leverages the unique Bragg peak for precise dose sculpting, allowing for high-dose target coverage concurrently with ultra-low dose to normal tissues (5). This advantage is critical for treating multifocal metastases. For example, photon radiotherapy often exceeds normal lung V20 constraints when irradiating multiple pulmonary lesions, significantly increasing pneumonitis risk. In contrast, proton therapy can confine the high-dose region tightly to the targets via optimized pencil beam scanning (6). However, current clinical evidence on the application of PBT in multifocal ACC pulmonary metastases remains very limited, particularly lacking quantitative analysis of long-term local control and organ-sparing efficacy. This article reports a case of refractory ACC with cervical recurrence and 16 bilateral pulmonary metastases. The patient experienced rapid progression despite multiple prior surgeries. Due to the large number and wide distribution of metastases, photon radiotherapy was deemed contraindicated. Based on a comparison of dose distributions, the patient was selected for staged proton therapy. This approach delivered radical doses (GTV 60–70 Gy(RBE)) simultaneously to the cervical and pulmonary metastases, achieving sustained remission. This case provides evidence to support the clinical application of PBT in complex metastatic ACC. Furthermore, it elucidates the physical principles and clinical value of PBT in overcoming the dose limitations of traditional radiotherapy.

## Case report

**Figure 1:**
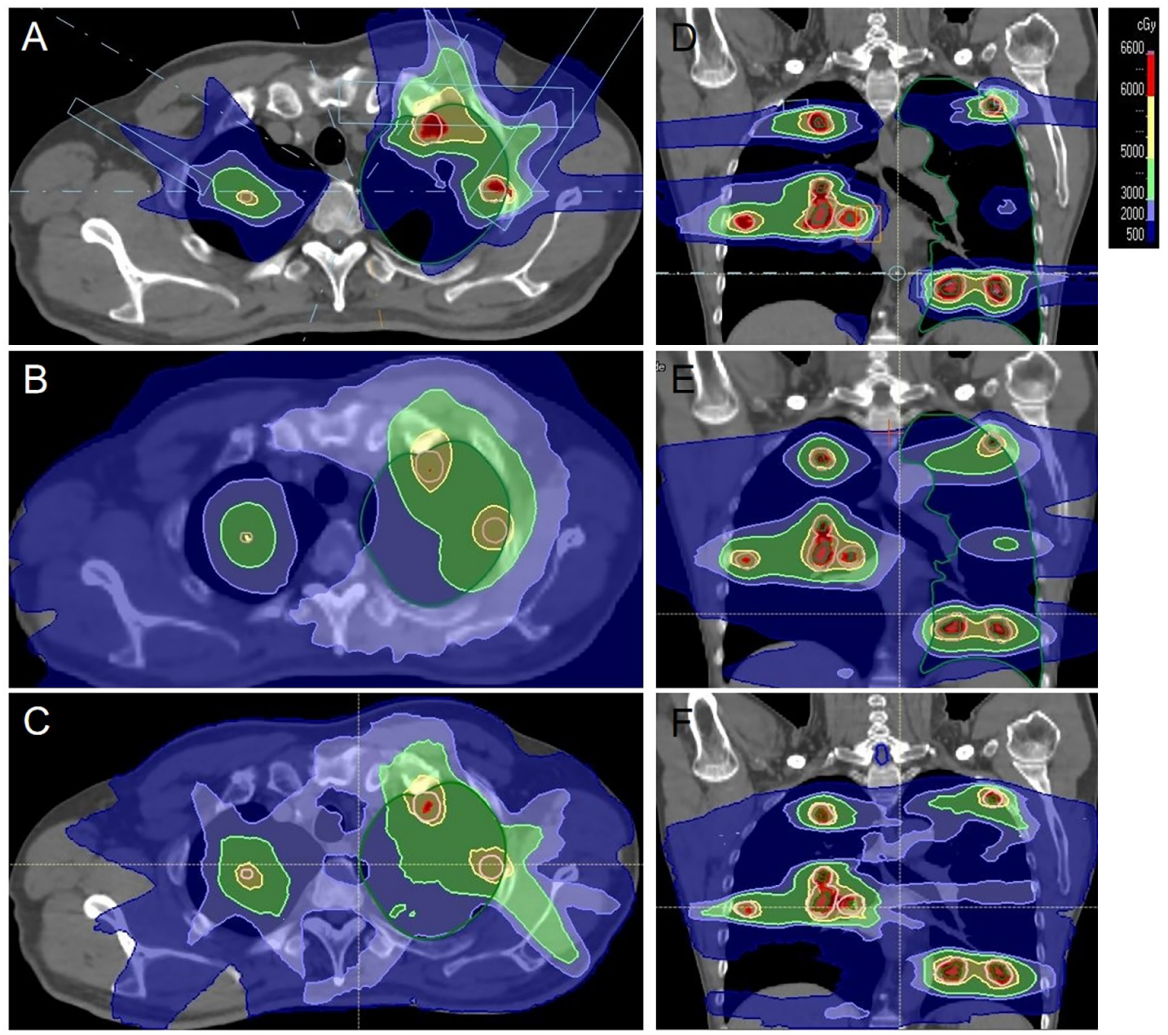
An example of the dose distribution for the proton and photon plans in axial and coronal planes. The colorwash was set from 500 to 6600Gy(RBE). **(A, D)** proton, **(B, E)** TOMO, **(C, F)** VMAT.

**Figure 2:**
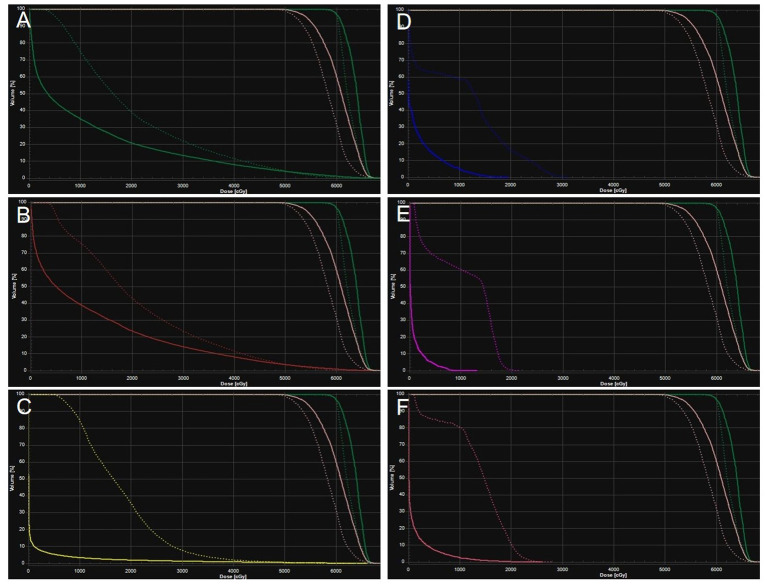
Averaged dose-volume histogram for protons (solid) and TOMO (dotted) for planning target volume (green and pink), left lung **(A)**, right lung **(B)**, heart **(C)**, spinal cord **(D)**, trachea **(E)**, esophagus **(F)**.

**Figure 3:**
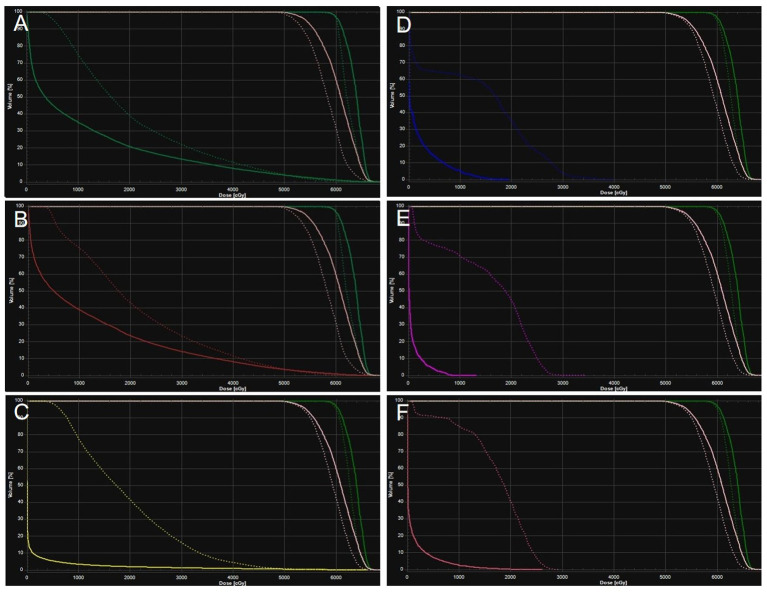
Averaged dose-volume histogram for protons (solid) and VMAT (dotted) for planning target volume (green and pink), left lung **(A)**, right lung **(B)**, heart **(C)**, spinal cord **(D)**, trachea **(E)**, esophagus **(F)**.

**Figure 4:**
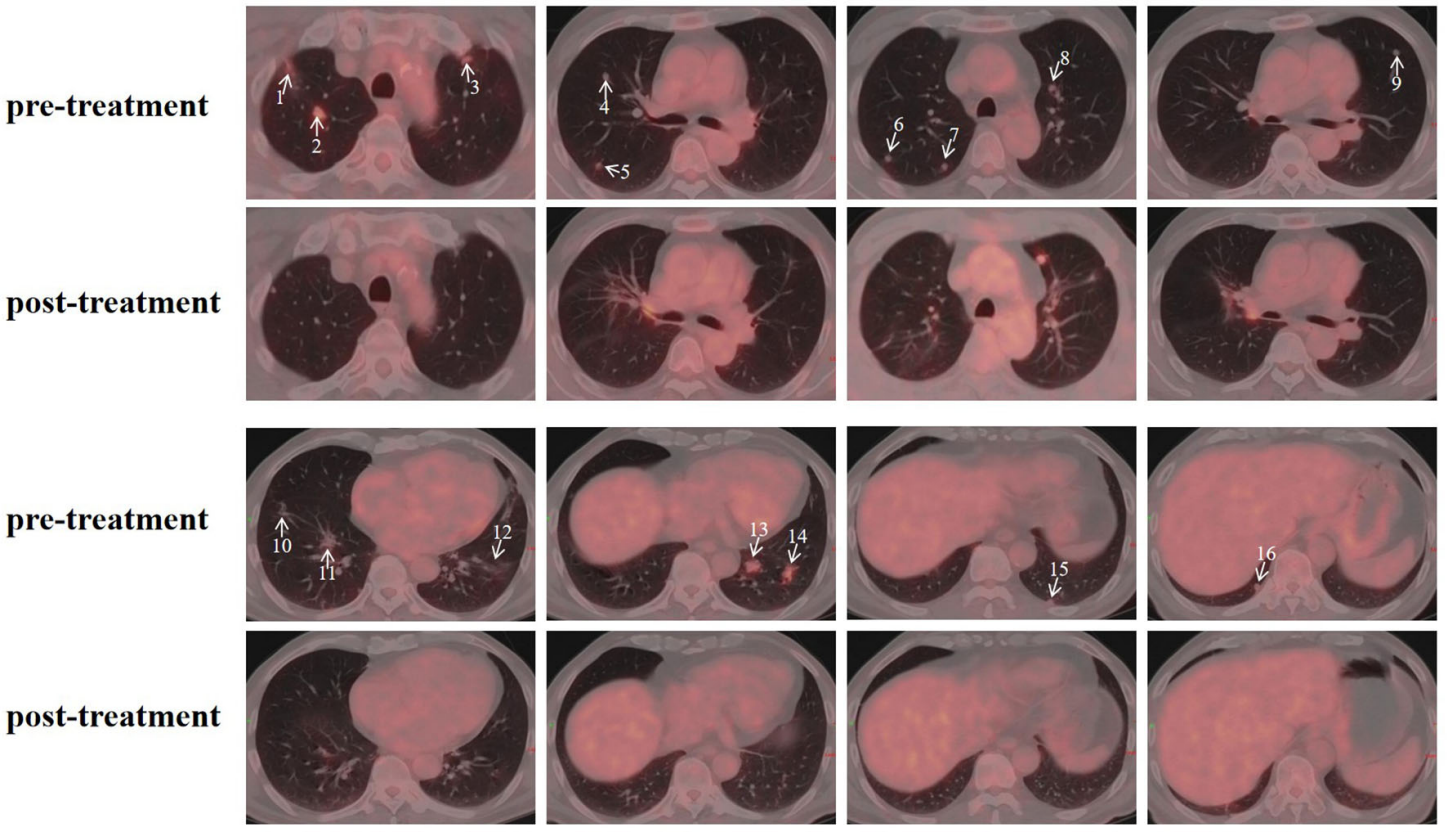
Pre- and post-treatment PET/CT images of pulmonary metastases. Arrows indicate the locations of the metastatic lesions, with numbers corresponding to the lesion IDs in **Supplementary Table**.

**Figure 5:**
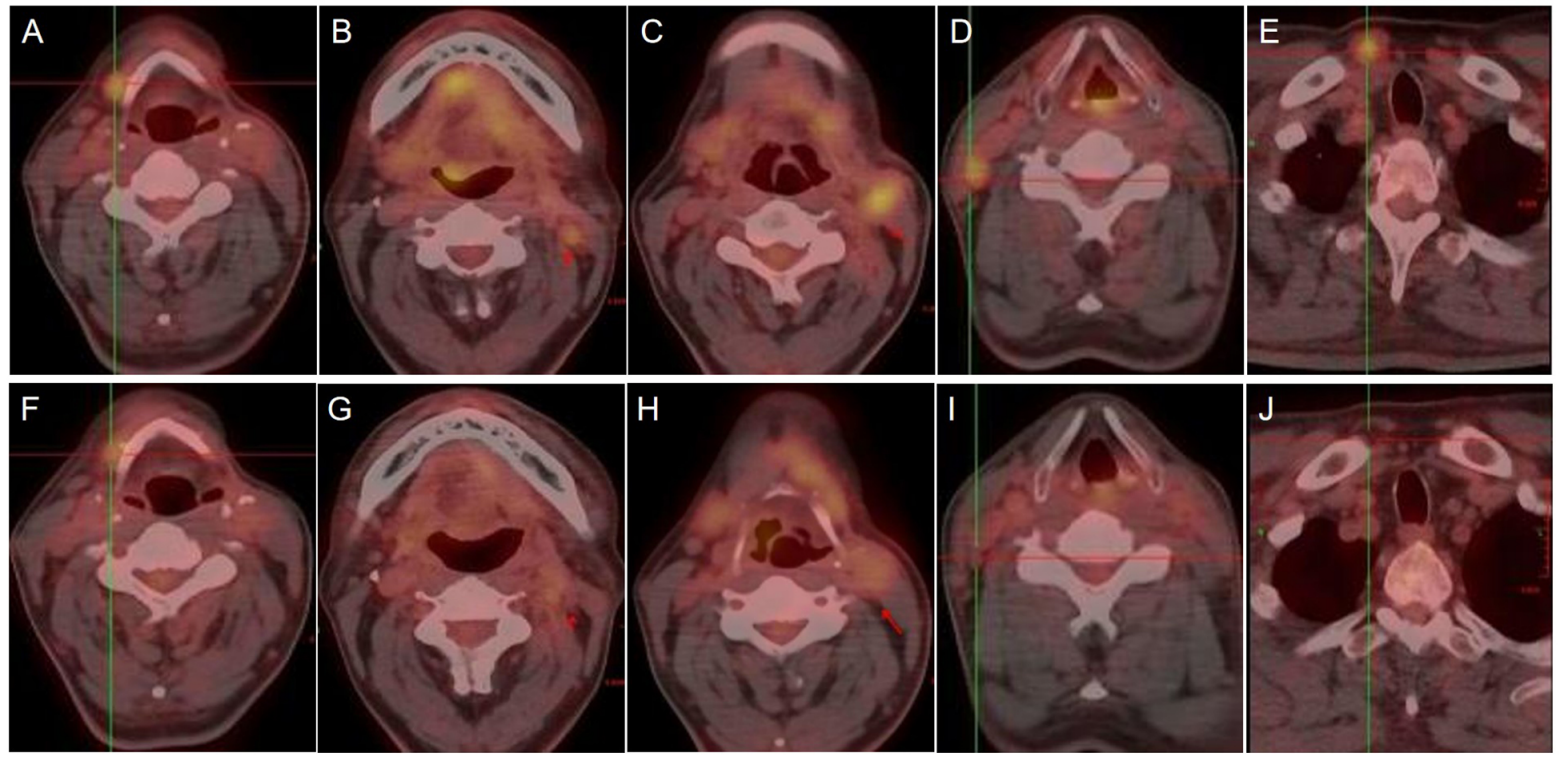
PET/CT images showing cervical lymph nodes before **(A-E)** and after **(F-J)** treatment with proton therapy.

The patient’s clinical timeline is summarized in **Table 1**, illustrating key milestones from diagnosis through prior treatments, proton therapy, and follow-up. A 54-year-old male patient initially presented in October 2019 with pharyngeal discomfort, revealing a left tongue base mass (1×2 cm, hard, fixed) and a left neck mass (medial to sternocleidomastoid, 4×2 cm, painless). On October 31, 2019, he underwent extended resection of the left tongue malignant tumor and left neck dissection. The final pathology confirmed adenoid cystic carcinoma of the left floor of mouth and tongue base, showing infiltrative growth, necrosis, and perineural invasion (+), with metastasis in 1 of 15 left upper deep cervical lymph nodes. No adjuvant radiotherapy/chemotherapy was administered postoperatively. In April 2020, a new painless left neck mass (1.5×1.0 cm) appeared and progressively enlarged. On August 4, 2020, selective left neck dissection revealed metastases in 5 of 15 lower deep cervical lymph nodes, consistent with ACC metastasis. By December 2020, recurrent masses had appeared in the left neck, accompanied by a floor-of-mouth mass and mild pain. On March 19, 2021, reoperation (left neck lymph node dissection and extended resection of the floor-of-mouth mass) confirmed ACC invasion into skeletal muscle and nerves in the submental lymph node and floor-of-mouth specimen. PET/CT (October 13, 2021) demonstrated bilateral cervical lymph node metastases (SUVmax 4.1) and 16 scattered bilateral pulmonary nodules (max diameter 16 mm, SUVmax 1.4). After multidisciplinary evaluation comparing TOMO (Helical TomoTherapy), VMAT (Volumetric Arc Therapy), and proton radiotherapy plans, photon radiotherapy (VMAT/TOMO) was deemed infeasible. **Figure 1** illustrates the dose distribution within the target volume for proton therapy, TOMO, and VMAT. For a quantitative comparison, **Figures 2**, **3** present the dose-volume histogram (DVH) curves of organs at risk, including the lungs, heart, spinal cord, trachea, and esophagus, for proton therapy versus TOMO and VMAT, respectively. PBT resulted in markedly decreased OAR metrics with equivalent target volume coverage. **Table 2** provides the dose parameters for the heart, lungs (including mean dose, V5, V20, V30), and spinal cord for proton therapy, TOMO, and VMAT respectively. Proton therapy was selected due to the high metastatic burden (16 pulmonary lesions and 5 cervical nodes), the absence of prior thoracic irradiation, and the need for both radical doses (BED≥70 Gy(RBE)) and stringent organ protection. This clinical profile fully aligned with the indications for proton therapy. The patient did not receive any systemic therapy (chemotherapy, targeted therapy, or immunotherapy), either concurrently or sequentially. A staged irradiation strategy was employed to treat the cervical and pulmonary metastases sequentially. Stage 1: Pulmonary Metastases (Oct 18 – Nov 2, 2021). Target Volume: GTV: 16 PET/CT-positive pulmonary nodules. CTV: GTV+5 mm margin. Dose Prescription: CTV: 50 Gy(RBE) in 15 fractions. GTV: 60 Gy(RBE) in 15 fractions. Technique: Pencil Beam Scanning (PBS), 70–230 MeV. Stage 2: Cervical Metastases (Commenced Nov 2, 2021). Target Volume: GTVnd: PET/CT-positive nodes. CTVnd: GTVnd+3 mm margin. CTV: Prophylactic region. Dose Prescription: GTVnd: 70 Gy(RBE) in 28 fractions. CTVnd: 66 Gy(RBE) in 28 fractions. CTV: 50.4 Gy(RBE) in 28 fractions. Technique: PBS with multi-field optimization. The Deep Inspiration Breath-Hold (DIBH) technique was used for all pulmonary treatments to manage respiratory motion and mitigate the interplay effect. Plans were created in the RayStation treatment planning system using a Monte Carlo dose calculation algorithm. Robust optimization was performed on the GTVs. The parameters were: 3.5% for range uncertainty and 3 mm (LR, AP)/5 mm (SI) for setup uncertainty. The clinical goal for the worst-case scenario was D97% ≥ 95% of the prescribed dose to the GTV. Efficacy was assessed by comparing PET/CT scans from April 21, 2023, with baseline scans from October 13, 2021. All 16 pulmonary metastases demonstrated significant regression or complete resolution, accompanied by reduction in metabolic activity (**Figure 4**). Concurrently, the cervical lymph nodes showed significant volume reduction and metabolic suppression (**Figure 5**). All observed toxicities (CTCAE v5.0) were acute and well-tolerated. The patient developed Grade 1 radiation dermatitis in the neck and Grade 2 oropharyngeal mucositis, characterized by moderate erythema and capillary dilation without confluent mucositis. Following pulmonary irradiation, Grade 1 radiation pneumonitis occurred after one month and resolved spontaneously without intervention. Notably, no cardiac, esophageal, or spinal cord toxicities were observed throughout the treatment and follow-up period.

**Table 1:** Timeline of patient’s clinical course from diagnosis to proton therapy.

Date (Year-Month)	Event description
2019-09	Initial diagnosis of Adenoid Cystic Carcinoma
2019-10	First surgery: Extended resection of left tongue base tumor and left neck dissection
2020-04	First post-operative recurrence: New mass in the left neck
2020-08	Second surgery: Selective left neck dissection
2020-12	Second post-operative recurrence: Recurrent left neck mass accompanied by floor-of-mouth mass
2021-03	Third surgery: Left neck lymph node dissection and extended resection of floor-of-mouth mass
2021-10	Third post-operative recurrence, with confirmed multiple metastases
2023-04	Follow-up and re-examination with PET-CT

**Table 2:** Dose metrics for planning treatment volume coverage and avoidance structures.

Metric	PBT	TOMO	VMAT
Heart mean (Gy)	1.3	17.87	19.25
Left lung mean (Gy)	11.38	20.44	21.29
Left lung V5 (%)	45.63	95.92	97.96
Left lung V20 (%)	20.86	39.12	40.19
Left lung V30 (%)	13.41	22.1	22.75
Right lung mean (Gy)	12.24	20.87	19.16
Right lung V5 (%)	50.11	92.86	83
Right lung V20 (%)	23.48	42.95	38.08
Right lung V30 (%)	14.19	23.42	21.82
Spinal cord Dmax (Gy)	17.29	30.44	39.26

## Discussion

The 16 bilateral pulmonary metastases presented a multicentric and scattered distribution. This anatomical presentation, combined with their proximity to critical organs, created a significant dosimetric challenge for conventional photon radiotherapy. Firstly, a fundamental dose conflict existed. Synchronously irradiating all 16 pulmonary nodules with SBRT would cause the normal lung V20 to exceed the safe threshold of 30%, thereby elevating the risk of radiation pneumonitis to approximately 40% (7). Secondly, IMRT could not achieve the dual goals of organ protection and radical dosing. Although better at sparing organs, it failed to deliver higher BED to multiple foci, severely limiting local control (8). Comparative DVH and dose cloud analyses of proton versus TOMO and VMAT plans in this patient clearly demonstrated this limitation. Conversely, proton therapy leveraged Bragg Peak physics and pencil beam scanning to overcome these limitations. It successfully delivered high radical doses to all targets—60 Gy(RBE) (BED 84 Gy(RBE)) to pulmonary GTVs and 70 Gy(RBE) (BED 87.5 Gy(RBE)) to cervical GTVnd—while simultaneously maximizing organ sparing (9). DVH analysis confirmed that doses to the lungs, heart, and esophagus remained substantially below QUANTEC constraints (10). This dosimetric advantage was reflected in the clinical outcome of only Grade 1–2 toxicities. Therefore, for extensive metastatic ACC, proton therapy overcomes the fundamental dose limitations of photon radiotherapy. It thereby provides a viable radical solution for traditionally “untreatable” cases.

Despite their typically indolent nature, multifocal ACC pulmonary metastases (such as the 16 in this case) respond poorly to systemic therapy, with response rates below 20% (11). The high-dose irradiation from proton therapy may overcome therapeutic resistance through two key mechanisms. First, its higher linear energy transfer (LET) enhances DNA damage by increasing double-strand breaks, which improves killing efficiency for hypoxic ACC cells by 30–50% (12). Second, the delivered dose of 70 Gy(RBE) can effectively disrupt the perineural invasion microenvironment, thereby reducing the risk of local recurrence (13). At the 18-month follow-up, pulmonary metastases in this case showed partial disappearance and sustained metabolic suppression in residual nodules. Our literature review confirms the role of proton in treating primary head and neck ACC and pulmonary oligometastases from various cancers, where it achieves excellent local control with minimal toxicity. A study on proton and carbon ion therapy for adenoid cystic carcinoma (ACC) reported 5-year rates of 63% for overall survival, 39% for progression-free survival, and 75% for local control. Even patients with T4 or unresectable disease benefited, achieving 5-year local control rates of 66% and 68%, respectively (14, 15). However, we found no specific previous studies on its application for multiple ACC pulmonary metastases, particularly with a high tumor burden like the 16 lesions in our case. Previous reports on pulmonary oligometastases rarely include such a high number of metastases (16). This case helps fill that gap, thereby providing initial evidence and supporting future multi-center research to better define the role of PBS-PT in this setting.

Building on this case’s success, we propose a screening framework for proton therapy in multifocal metastatic ACC: For confirmed ACC patients with multifocal metastases, first analyze lesion number and distribution. If ≥5 metastases exist, or lesions overlap organs at risk (e.g., pulmonary metastases adjacent to heart/esophagus), deem photon radiotherapy contraindicated and prioritize proton therapy. For ≤4 limited metastases without OAR overlap, photon techniques (SBRT/IMRT) remain suitable. Thus, proton therapy is prioritized for highly complex metastases (multifocal or in anatomically high-risk zones), while low-burden localized metastases may still utilize conventional photons.

Despite its superior dosimetric outcomes, several technical and practical challenges associated with proton therapy should be mentioned. Firstly, the interplay effect between pencil beam scanning (PBS) and tumor motion posed a significant concern for the multiple pulmonary metastases. We mitigated this risk through a comprehensive motion management strategy including 4D-CT simulation and respiratory gating technique. Furthermore, a strategy of multiple fields and a volumetric repainting technique was applied for each fraction (17). Secondly, sensitivity to anatomical changes and inherent range uncertainty of proton required diligent management. Throughout the treatment process, we employed daily cone-beam CT (CBCT) for precise image guidance and anatomical monitoring. Additionally, the treatment plan was optimized using robust optimization to account for a 3.5% range uncertainty and setup uncertainties of 3 mm (LR, AP)/5 mm (SI). Finally, the substantial cost and increased resource utilization of proton therapy cannot be overlooked (18). Techniques like respiratory gating and complex multi-field plans further extend treatment times. However, for selected complex cases like this, the higher initial cost must be weighed against the potential for long-term benefit. In young patients with multifocal, radio-resistant disease, achieving durable local control while reducing severe toxicity can potentially offset long-term costs associated with disease progression or complication management (19).

Current median follow-up remains short, requiring extension beyond 5 years to assess long-term control and late toxicity. Furthermore, findings from a single case necessitate validation via multicenter prospective clinical studies. Future advancements like FLASH radiotherapy and PBS optimization may further reduce treatment costs (20).

## Data Availability

The datasets presented in this study can be found in online repositories. The names of the repository/repositories and accession number(s) can be found in the article/**Supplementary Material**.
