# Supplementary material for: Proton therapy achieves high-dose tumor control with organ preservation in complex metastatic adenoid cystic carcinoma: a case report of a refractory patient with 16 pulmonary metastases
Source: Front Oncol. 2025 Nov 6;15:1669420. doi: 10.3389/fonc.2025.1669420 (PMC12631414; doi:10.3389/fonc.2025.1669420)
Supplement: Supplementary file 1 [file Table1.docx]

**Supplementary Table 1**

| Lesion | Short axis(mm) | | SUV | |
| --- | --- | --- | --- | --- |
|  | Pre | Post | Pre | Post |
| A | 11 | 6 | 3.1 | 1.4 |
| B | 10 | 2 | 2.8 | 1.1 |
| C | 16 | 10 | 4.1 | 1.2 |
| D | 11 | 5 | 2.7 | / |
| E | 14 | 2 | 3.1 | 0.8 |

Treatment response of cervical lymph node metastases

Short-axis diameter and SUVmax values of measured cervical lymph node metastases at baseline (Pre) and after (Post) proton therapy. A slash (/) indicates complete radiological/metabolic resolution of the lesion.

**Supplementary Table 2**

| Lesion | Long axis(mm) | | SUV | |
| --- | --- | --- | --- | --- |
|  | Pre | Post | Pre | Post |
| 1 | 6 | 2 | 0.8 | 0.2 |
| 2 | 13 | 6 | 1.1 | 0.3 |
| 3 | 9 | 2 | 0.8 | 0.3 |
| 4 | 7 | 2 | 0.5 | / |
| 5 | 8 | 2 | 0.7 | / |
| 6 | 7 | / | 0.5 | / |
| 7 | 7 | / | 0.6 | / |
| 8 | 8 | / | 0.6 | / |
| 9 | 7 | / | 0.5 | / |
| 10 | 10 | / | 0.5 | / |
| 11 | 15 | 8 | 0.9 | 0.2 |
| 12 | 13 | 5 | 0.9 | 0.2 |
| 13 | 15 | 3 | 1.2 | / |
| 14 | 16 | 4 | 1.4 | / |
| 15 | 8 | / | 0.6 | / |
| 16 | 7 | / | 0.7 | / |

Treatment response of pulmonary metastases

Long-axis diameter and SUVmax values of all 16 pulmonary metastases at baseline (Pre) and after (Post) proton therapy. A slash (/) indicates complete radiological/metabolic resolution of the lesion.
